# Screening Emotions in Adolescents Receiving Care at the Hospital for mild Traumatic Brain Injury (SEARCH-mTBI): protocol for a multicenter observational study

**DOI:** 10.1186/s12887-026-06788-5

**Published:** 2026-04-07

**Authors:** Daniel K. Nishijima, Nathan Kuppermann, Stacy J. Suskauer, Kristy B. Arbogast, Mohamed Badawy, T. Charles Casper, Andrea T. Cruz, Daniel J. Corwin, Tiffani J. Johnson, Elizabeth D. Rosenthal, Stephanie M. Ruest, Daniel J. Tancredi, Danny G. Thomas, Mark R. Zonfrillo, Beth S. Slomine, the SEARCH-mTBI Study Group

**Affiliations:** 1 Department of Emergency Medicine, University of California Davis School of Medicine, 4150 V Street, PSSB Suite 2100, Sacramento, CA 95817 USA; 2 Departments of Pediatrics and Emergency Medicine, George Washington University School of Medicine and Health Science and Children’s National Hospital, Washington, USA; 3 Departments of Physical Medicine & Rehabilitation and Pediatrics, Kennedy Krieger Institute and Johns Hopkins University School of Medicine, Baltimore, MD USA; 4 Division of Emergency Medicine, Children’s Hospital of Philadelphia, Philadelphia, PA USA; 5 Department of Pediatrics, University of Texas Southwestern Medical Center, Dallas, TX USA; 6 Department of Pediatrics, University of Utah School of Medicine, Salt Lake City, UT USA; 7 Department of Pediatrics, Baylor College of Medicine, Houston, TX USA; 8 Kennedy Krieger Institute, Johns Hopkins University School of Medicine, Baltimore, MD USA; 9 Departments of Emergency Medicine and Pediatrics, Warren Alpert Medical School of Brown University, Providence, RI USA; 10 Department of Pediatrics, Medical College of Wisconsin, Milwaukee, WI USA; 11 The Graduate Center, City University of New York, New York, NY USA

**Keywords:** Mild traumatic brain injury, Adolescents, Mental health, Protocol, Observational study, Disparities, Prediction model

## Abstract

**Background:**

Mild traumatic brain injury (mTBI) is a common pediatric condition with growing recognition of its impact on mental health. While most children recover within weeks of the injury, a subset experience persistent emotional symptoms, including anxiety and depression. Early identification of youth at risk for adverse mental health outcomes is essential for timely intervention. Our primary study objective is to develop a robust clinical prediction tool to identify children at risk for adverse mental health outcomes after mTBI.

**Methods:**

We will conduct a multicenter prospective observational cohort study enrolling patients aged 11–17 years who present to the emergency department (ED) with mTBI within 3 days from injury. We will enroll consecutive patients into separate derivation and validation cohorts using different enrolling sites. Baseline data, including pre-injury mental health status, and clinical variables will be collected from patients, caregivers, and ED clinicians. We will collect validated measures during the acute period (1 to 2 weeks), 1-month, and 3-months post-injury including: the Generalized Anxiety Disorder-7 (GAD-7), Patient Health Questionnaire-8 (PHQ-8), Pediatric Quality of Life Inventory (PedsQL), Rivermead Post-Concussion Symptoms Questionnaire, and the Strengths and Difficulties Questionnaire (SDQ). Our primary outcome is a composite measure of clinically significant new or worsening in anxiety or depression at 1 or 3 months. Secondary outcomes include persistent post-concussive symptoms, reduced quality of life, and new behavioral or emotional difficulties. We will analyze associations between baseline factors and outcomes to derive and validate a clinical prediction tool.

**Discussion:**

Our study will provide a comprehensive assessment of mental health and other outcomes following mTBI in adolescents and identify predisposing factors for adverse outcomes. Our findings will contribute to the development of a clinical prediction tool to support early identification and referral for at-risk youth. Study results may inform post-injury care pathways and targeted interventions aimed at improving long-term recovery.

**Trial registration:**

ClinicalTrials.gov Identifier: NCT06370520. Registered March 20, 2024. The study protocol has been approved by the Institutional Review Boards at all participating sites.

**Supplementary Information:**

The online version contains supplementary material available at 10.1186/s12887-026-06788-5.

## Background

Mental health sequelae are common in adolescents after mild traumatic brain injuries (mTBI). As defined by the Centers for Disease Control and Prevention (CDC), mTBI includes concussion, an acute brain injury resulting in neurological symptoms. These injuries are associated with a Glasgow Coma Scale (GCS) score of 13 to 15 in association with confusion or disorientation, loss of consciousness, amnesia, seizures, or other signs or symptoms of head injury [1, 2]. mTBI in children is a major public health concern in the United States (US) and globally and is associated with decreased health-related quality of life following injury [3–5]. After youth mTBI, cognitive and functional activity tends to return to baseline within three months [6]. However, neuropsychiatric recovery is more complex. In prior studies, 25–50% of children with mTBI had substantial mental health sequelae after head injury, with depression and anxiety the most common [7–10]. Anxiety and depression may be a result of injury or may develop or worsen due to lingering symptoms or restrictions after injury [11]. 

Identification of adolescents at risk for mental health sequelae after mTBI is a key knowledge gap. The CDC Guidelines on the Diagnosis and Management of mTBI among Children, published in 2018, recommend that health care professionals screen for known risk factors for persistent symptoms in children with mTBI [2]. Prior studies have identified risk factors for mental health sequelae after mTBI [12–21]. These factors include socioeconomic status, severity of injury, and pre-existing and family history of psychiatric disorders. Unfortunately, most of these studies include small cohorts (*n* < 200) and are thus unable to provide sufficient evidence for a robust and precise prediction tool that clinicians can use to risk stratify children after mTBI.

Because no single factor is strongly predictive of mental health sequelae, identifying children at risk is challenging. One validated clinical prediction tool identifies children with mTBI at risk for persistent concussive symptoms at 28-days; [22] however, there are no clinical prediction tools that specifically address risk factors for mental health sequelae [2]. As such, prior studies have demonstrated that 31–78% of children with mTBI who have new or worsening mental health concerns are not receiving appropriate mental health care [23–25]. The primary objective of our study is to develop a robust clinical prediction tool to identify children at risk for adverse mental health outcomes after mTBI. This tool will fill an important gap in the field and assist health care providers in managing adolescents with mTBI.

## Methods

### Study design and setting

We will conduct a multicenter, prospective observational study of adolescents with mTBI to develop a clinical prediction tool that identifies those at risk for adverse mental health outcomes. The study will be conducted in six emergency departments (EDs) across the United States. All sites are American College of Surgeons (ACS) verified Level I pediatric trauma centers.

Participants will be enrolled into two sequential cohorts: a derivation cohort at four sites and a validation cohort at three sites. The clinical coordinating center will enroll in both the derivation and validation cohorts to monitor enrollment throughout the study. This design constitutes a true external validation, as the prediction tool will be derived and tested on an independent patient population than the derivation cohort. External validation is considered a more rigorous approach than internal validation, which uses the same cohort for both derivation and testing.

The clinical prediction tool will first be derived using only the derivation cohort. Investigators will remain blinded to the data from the validation cohort until the prediction tool has been fully developed. The finalized tool will then be validated in the second, independent cohort.

This study will follow the STROBE (Strengthening the Reporting of Observational Studies in Epidemiology) guidelines, and a completed checklist will be included with manuscript submission (see Additional File 1) [26]. 

### Participants

We will enroll adolescents aged 11 to 17 years who present to the ED within 72 h of mTBI. We will use the CDC definition of mTBI, which is an acute brain injury resulting in neurological symptoms such as confusion or disorientation, headache, nausea, loss of consciousness, amnesia, seizure, focal neurological signs or symptoms, and/or traumatic intracranial abnormalities on computed tomography (CT) or magnetic resonance imaging (MRI), in association with Glasgow Coma Scale (GCS) scores of 13 to 15. Consistent with CDC precedent, this definition includes commonly used terms such as “concussion” or “minor head injury” [13]. Participants may include patients with neuroimaging findings indicative of traumatic abnormalities (e.g., intracranial hemorrhage, diffuse axonal injury, skull fractures), as these are considered risk factors for later mental health sequelae [9, 11, 27]. However, neuroimaging is not required for study enrollment.

Patients will be excluded if any of the following apply:

- Presentation to the ED more than 72 h after injury.
- TBI requiring emergent neurosurgical intervention at enrollment.
- Other injuries requiring emergent surgery at enrollment.
- Inability of the patient or parent to accurately complete study questionnaires due to preexisting functional limitations (e.g., severe intellectual development).
- Previous known enrollment into the study.
- Inability of the patient or parent to speak English or Spanish.

### Participant screening and consent

Injured children aged 11 to 17 years presenting to the ED will be screened by either research coordinators or treating clinicians. For all patients who meet the inclusion criteria, we will complete an eligibility case report form to document inclusion and exclusion criteria. These data will inform the STROBE-compliant flowchart [26]. To ensure that the study sample is representative of the broader mTBI population, electronic health records for injured children aged 11 to 17 years seen in the ED will be routinely reviewed throughout the enrollment period. If a potentially eligible patient is not approached for enrollment, the research coordinator will complete a brief form capturing basic demographic and clinical information available retrospectively. This process will support assessment of potential enrollment bias.

Eligible patients will be approached by a research coordinator or treating clinician. We will obtain verbal informed consent from a parent or caregiver. Parents or caregivers will receive a one-page information sheet outlining the study purpose, procedures, risks and benefits, compensation, and contact details for research staff and the institutional review board. Information sheets will be available in both English and Spanish. If a parent or caregiver declines participation, the reason for non-participation will be recorded on the eligibility case report form, and the patient will not be enrolled. Upon enrollment, parents or caregivers will electronically provide their contact information for questionnaire completion.

### Validated questionnaires

We have selected validated questionnaires based on availability in English and Spanish languages, feasibility of completion, and recommendations by the CDC, which include the NIH TBI common data elements [28]. 

Generalized Anxiety Disorder-7 (GAD-7): The GAD-7 questionnaire is a widely used self-report measure to screen for anxiety symptoms and severity. It consists of seven questions related to anxiety with responses scored from 0 (no symptoms) to 3 (symptoms nearly every day). It has been validated as a diagnostic tool for generalized anxiety disorder in adults and adolescents, panic disorder, social phobia, and post-traumatic stress disorder (70–90% sensitivity and 80–90% specificity across disorders) [29–32]. The GAD-7 has been used as an anxiety measure for prior mTBI studies in adolescents [33]. A cutoff of 10 points or higher (maximum is 21 points) is frequently used to determine a higher risk for disability and functional impairment. A change of 4 points on the GAD-7 is the minimal clinically important difference [34]. Higher GAD-7 scores correlate with worse disability and functionality. The questionnaire is available in English and Spanish and takes two minutes to complete.

Patient Health Questionnaire-8 (PHQ-8): The PHQ-8 is the self-report major depressive disorder module of the full PHQ and is used to diagnose depression and grade severity of symptoms in both medical and mental health settings [35]. It consists of 8 questions related to depression and responses are scored from 0 (“no symptoms”) to 3 (“symptoms nearly every day”). It is similar to the PHQ-9, which has been validated in adults and adolescents [36, 37], and has been used to assess depression in prior mTBI studies in adolescents [33, 38], but does not include a question about potential suicidality (“How often have you had thoughts that you would be better off dead, or thoughts of hurting yourself in some way”) [37, 39]. The PHQ-8 is used frequently in place of the PHQ-9 in large studies because it is often infeasible to provide appropriate psychiatric intervention should the potential suicidality question be answered positively [40]. The PHQ-8 has similar discriminatory capacity as the PHQ-9 [40]. A cutoff of 10 points or more (out of 24) would correlate to a moderate or higher severity of depression symptoms and has excellent sensitivity and specificity in various populations [41–43]. The minimal clinically important difference is a change of 5 points on the PHQ-8 [44]. Higher PHQ-8 scores are associated with decreased functional status and increased symptom-related difficulties, sick days, and healthcare use [42]. The PHQ-8 is available in English and Spanish and takes less than two minutes to complete. 

The Rivermead Post-Concussion Symptoms Questionnaire (Rivermead): This self-report questionnaire is a frequently used and validated measure to evaluate for symptoms after mTBI and is a NIH core common data element for symptoms [45–47]. Symptoms are rated from 0 to 4, where 0 = no symptoms at all; 1 = no more of a problem than before injury; 2 = a mild problem compared to before injury; 3 = a moderate problem compared to before injury; and 4 = a severe problem compared to before injury. The Rivermead is available in English and Spanish and takes about five minutes to complete.

Pediatric Quality of Life Inventory (PedsQL): The PedsQL has been reported in more than 850 publications and measures health-related quality of life in healthy children and adolescents as well as those with acute and chronic health conditions [48]. There are age-appropriate versions for the PedsQL, including 8–12 years and 13–18 years in the adolescent age range, and age-appropriate versions can be combined for analysis. The PedsQL Generic Core scales are comprised of 23 questions that measure core dimensions of health as delineated by the WHO. The four multidimensional scales include: physical (8 items), emotional (5 items), social (5 items), and school functioning (5 items). The PedsQL is developmentally appropriate, reliable, and valid (distinguishes between healthy children and children with acute and chronic health conditions). For TBI, the PedsQL reliably differentiates between TBI severity in children [49]. The PedsQL is measured as a continuous score that ranges from 0 to 100, and a clinically meaningful difference is indicated by a 4.5-point change on the PedsQL scale [50]. The PedsQL is recommended by the NINDS Common Data Elements TBI Outcomes Workgroup as a core quality-of-life measure post-TBI and a primary measure of global outcome [51]. It is available in English and Spanish and takes approximately five minutes to complete. We will be collecting both self and proxy PedsQL reports.

Strengths and Difficulties Questionnaire (SDQ): We will use the SDQ standardized caregiver-report questionnaire to measure emotional symptoms, conduct problems, inattention/hyperactivity, and peer relationship problems. The 20 questions that make up these 4 scales are used to calculate a composite “total difficulties” score [52]. Each of the items are rated as “not true”, “somewhat true”, and “certainly true”. For this study, we will use the emotional distress and hyperactivity/inattention sub-scales (5-items each) to assess for parent-reported psychological distress and hyperactivity/inattention of the patient. We will use a cutoff score of 5 or more points to define psychological distress and 6 or more points to define hyperactivity/inattention [52]. These cutoff scores have a population base rate of less than 10%. The SDQ is a core common data element for psychological status in TBI. The SDQ is available in English and Spanish and takes less than five minutes to complete.

Early Physical Activity/Return to School Questionnaire: Early physical activity is defined as any level of physical activity, other than no activity, seven days after the index ED visit [12]. Return to physical activity within seven days of injury is associated with lower rates of post-concussive symptoms [12, 18]. We will assess for this early physical activity using a single self-report question categorized as no activity (e.g., physical rest), light to moderate aerobic activity (e.g., walking, swimming, stationary cycling, or jogging); heavy aerobic activity (e.g., running) or non-contact training drills (e.g., passing drills with teammates); and full-contact practice or games (e.g., normal training and return to competition).

Similarly, early return to school (defined as < 3 school days missed) is associated with lower post-concussive symptom burden at 14 days [53]. We will assess early return to school using a single self-report question 1 to 2 weeks after the index ED visit, categorized as early return to school or late return to school (3 or more school days missed) [53]. 

Mental Health Utilization Questionnaire: We will assess the patient’s mental and behavioral health treatment based on previously developed methodology, assessing parent-reported treatment and services used and unmet needs [24, 25]. This questionnaire is available in English and Spanish and takes less than two minutes to complete.

### Outcomes

Our primary outcome is the presence of mental health sequelae, defined as a composite measure of clinically significant new or worsening in anxiety or depression 1 to 3 months following mTBI. This will be assessed using the GAD-7 and the PHQ-8. For the GAD-7, a clinically meaningful increase is defined as a rise of 4 or more points from baseline (pre-injury retrospective report) at either the 1- or 3-month follow-up. For the PHQ-8 a clinically meaningful increase is defined as a rise of 5 or more points from baseline at either the 1- or 3-month follow-up [44]. Thus, the primary outcome is binary and is considered met if a participant demonstrates a clinically significant increase on either the GAD-7 or PHQ-8 at 1 or 3 months post-injury.

We will assess secondary outcomes using data collected at the 1- and 3-month follow-up timepoints to further characterize the study cohort. While the primary outcome focuses on mental health sequelae, mTBI may impact other domains of functioning. These secondary outcomes will help provide a more comprehensive understanding of the broader consequences of mTBI and the context of mental health changes. We will evaluate the association between each secondary outcome and the predicted risk of the primary outcome based on our risk-stratification model. Additionally, we will explore potential risk factors for each secondary outcome, which include:

Decline in quality of life: Evaluated as a binary variable, a decline will be considered present if there is a decrease of 4.5 points or more in the PedsQL score at either the 1- or 3-month follow-up compared to baseline.

Persistent mTBI symptoms: Evaluated as a binary variable, persistent symptoms will be scored using the Rivermead. This outcome will be met if the participant reports a score of 2 or higher on any three symptoms at either follow-up point.

New emotional/behavioral difficulties or hyperactivity/inattention: These will be assessed using the SDQ. New emotional/behavioral problems will be defined as a 5-point or greater increase on the emotional symptoms subscale. New hyperactivity/inattention problems will be defined as a 6-point or greater increase on the corresponding subscale, compared to baseline scores, at either the 1- or 3-month follow-up.

### Data collection procedures

Baseline: Following enrollment, we will collect baseline data from the patient, parent or caregiver, and ED clinician. This will include contact information, demographic details, medical history, and relevant clinical variables. Accounting for pre-injury functioning is essential to identify preexisting mental and behavioral health symptoms and to distinguish them from new or worsening symptoms following injury -- a standard practice in post-injury assessment [22, 54]. Therefore, we will gather baseline mental health and related data reflecting the patient’s status prior to the mTBI. This will include the GAD-7, PHQ-8, PedsQL, social determinants of health, Rivermead, and mental health service utilization history (Table 1).

**Table 1:** Schedule of evaluations

Construct	Measure	Respondent	Baseline	Acute Period	Outcome Period	
			ED ^a^	1 to 2 weeks ^a^	1-month ^a^	3-months ^a^
Eligibility	CRF	RC	x			
Contact information	CRF	Parent	x			
ED clinical variables	CRF	Physician	x			
Medical record variables ^b^	CRF	RC	x			
Family demographics and medical history	CRF	Parent	x			
Anxiety	GAD-7 ^d^	Child	x		x	x
Depression	PHQ-8 ^d^	Child	x		x	x
Health related quality of life	PedsQL^d^	Child and Parent	x		x	x
Behavioral screening	SDQ ^d^	Parent	x		x	x
mTBI symptoms	Rivermead ^d^	Child	x ^c^	x^c^	x^c^	x^c^
Mental health utilization	Questionnaire ^d^	Parent	x	x	x	x
Early physical activity/Return to school	Questionnaire ^d^	Child		x		

*Abbreviations*: *CRF *Case review form, *GAD-7* Generalized Anxiety Disorder-7, *PHQ*-*8 *Patient Health Questionnaire-8, *SDQ *Strength and Difficulties Questionnaire, *Rivermead *Rivermead Post Concussion Symptom Questionnaire

^a^Collected via automated digital surveys or the Central Outcomes Center at the Kennedy Krieger Institute

^b^﻿RC completion of medical record variables will be completed after hospital discharge

^c^Current symptoms rated compared to pre-injury baseline symptoms

^d^Time to completion (in minutes): GAD-7 (2), PHQ-8 (2), Rivermead (5), PedsQL (5), SDQ (5), Mental health utilization (1), Early physical activity/return to school (1)

Follow up: Automated digital surveys will be sent to the parent or caregiver for the acute period (1 to 2 weeks after the index ED visit), 1-month follow-up, and 3-month follow-up. The Central Outcomes Center at the Kennedy Krieger Institute will monitor survey completion and if necessary, will contact parents or caregivers to complete the follow up surveys. After completion of each baseline and follow up survey, patients and parents or caregivers will be emailed a gift card of their selection using the Tango gift card service. In cases where the parent or caregiver does not have an email address, the Central Outcomes Center will mail gift cards to the home address of the patient and parent or caregiver.

### Analysis

We will select candidate variables a priori based on prior studies and clinical expertise. Variables will include patient demographics, injury characteristics, pre-injury mental health diagnoses, treatment, early physical activity, and persistent mTBI symptoms (at 1 to 2 weeks). We will elicit clinical expertise during the coding of the clinical prediction steps, including rules for addressing item-level nonresponse. We will evaluate the impact of individual predictors by estimating odds ratios with 95% confidence intervals.

We will rely primarily on Classification and Regression Tree (CART) analyses using binary recursive partition in addition to multivariable logistic regression for model development. For the derivation cohort, feature selection will separately use both elastic net regularization and bootstrapped backward elimination models, with selections compared to determine the model specification for estimating model parameters. We will derive a low-risk component of the risk stratification model using binary recursive partitioning. Recursive partitioning allows for the inclusion of patients with missing predictors by substituting “surrogate” variables that partition patients as the missing variables would. Therefore, patients with missing observations for one or more predictors are not necessarily dropped from the analysis (as they often are in logistic regression analysis). While this property is attractive, it may also lead to bias if there is excessive use of surrogate variables. Therefore, if more than 20% of the data are missing for any variable (consistently across all sites), that variable will be excluded from consideration into the model. This proposed restriction is also important from a practical standpoint, because if a frequently missing variable were part of the prediction rule, the clinician would be unclear about what to do with patients missing this information. We will monitor missingness throughout the study to allow for training of study personnel, if necessary and appropriate. In the construction of the decision tree, we will assign misclassification “costs” to specific misclassification errors. We will also enter sites into the analysis to explore whether any site exerts disproportionate influence in the generation of the model.

To complement the low-risk component of the risk stratification model created by recursive partitioning, we will use the same candidate variables to conduct multivariable logistic regression analyses to derive a risk score model for those patients identified as having greater than low risk (i.e. those who do not meet low-risk criteria). We will first conduct single variable logistic regression to identify all variables that have any association with the outcome.

Internal cross-validation methods will be used to refine the model. Following the development of the final model from the derivation cohort, we will use the Framingham Study method to translate logistic regression estimates into simple risk scores. Performance of the resulting risk-stratification models (original and simplified) will be validated in the validation cohort [55]. Model performance will include assessments of discrimination and calibration. Finally, we will also use random forests to derive a prediction algorithm for mental health sequelae. Many trees are independently grown using recursive partitioning on bootstrapped samples of observations. The predictor at each node in each tree is selected from a random subset of predictors and trees are grown without pruning.

Secondary outcomes will be analyzed using descriptive statistics to further characterize the study population. We will analyze how the primary risk model prediction is associated with secondary outcomes. For this analysis, we will use the combined derivation and validation cohorts.

### Sample size

We specified that a useful clinical prediction rule would have a true discriminatory capacity, as measured by the area under the receiver operating characteristic (AUROC) curve, of at least 80%. To account for the large number of candidate variables in model derivation, we used variance inflation factors based on chi-square non-centrality parameters to convert actual sample sizes into effective sample sizes. We conservatively assume that at least 25% of the enrolled patients will experience the primary outcome [7–10]. A derivation cohort of at least 1,400 patients would provide approximately 350 patients with mental health sequelae, allowing for more than 35 predictor degrees of freedom. The planned validation sample size of 1080 patients (accounting for loss to follow-up) provides adequate precision for estimates of discrimination performance. For example, at a threshold of 90% sensitivity, the 95% confidence interval for sensitivity will have a size of approximately ± 3.8%.

### Missing data

The primary and secondary outcomes will be collected at two time points (1- and 3-month follow-up) reducing the risk of loss to follow-up. Due to the multiple follow-up times, we anticipate low missingness rates for the primary and secondary analyses. Patients who withdraw from the study or are lost to follow-up will have all available data used in the analysis. If a substantial number of patients are withdrawn or lost to follow-up, we will review and compare baseline characteristics to patients not withdrawn or lost, to assess empirically if these patients differ from those remaining in the study. The primary and secondary regression analyses will use complete case analyses if no more than 5% of cases are dropped from the analysis due to missing predictor variable data.

If missing data results in dropping more than 5% of cases for validation purposes, we will use regression-based multiple imputation within a chained equations framework. We will use standard regression methods for missing primary and secondary outcomes to use in imputation models, using bootstrapped stepwise variable selection to select predictors. We will impute and analyze 10 datasets. We will increase that number if the efficiency of the imputed datasets is less than 0.99. Following standard multiple imputation steps, we will estimate parameters and corresponding covariance matrices separately for each imputation dataset and then pool the results.

## Discussion

Our prospective cohort study aims to evaluate the mental health sequelae of mTBI in children and adolescents, with a focus on identifying early predictors of post-injury anxiety and depression. While much of the pediatric mTBI literature has focused on persistent post-concussive symptoms such as headaches or dizziness, there is growing recognition of the frequency and importance of emotional and behavioral outcomes following mTBI. Our study addresses this gap by incorporating validated mental health assessments at multiple time points and by analyzing their relationships with baseline characteristics.

Although most children with mTBI recover within a few weeks, a substantial proportion experience persistent symptoms, including emotional and behavioral difficulties, that may affect their functioning and quality of life [56–60]. These include elevated rates of anxiety, depression, and post-concussive symptoms for months following injury, even among children without prior psychiatric diagnoses [8, 61, 62]. For example, Zemek et al. found that 30% of children had persistent post-concussion symptoms at 28 days, and emotional symptoms were among the most commonly reported [22]. Other longitudinal studies have demonstrated that early symptom burden—particularly emotional and cognitive symptoms—may predict longer-term mental health difficulties [63–65]. However, limitations of previous work include small or homogeneous samples, retrospective designs, and inconsistent use of validated tools to assess changes over time. Moreover, few studies have rigorously assessed pre-injury mental health status, making it difficult to distinguish new-onset symptoms from preexisting conditions. Our study addresses these gaps by prospectively tracking a diverse cohort, using validated measures, and systematically capturing pre-injury functioning to better interpret post-injury changes.

The primary outcome—a composite measure of clinically significant worsening in anxiety or depression using the GAD-7 and PHQ-8—will allow us to capture meaningful mental health changes in the months following injury. By anchoring this outcome to minimal clinically important differences, we aim to ensure clinical relevance and interpretability. In addition, the inclusion of secondary outcomes such as persistent mTBI symptoms, quality of life changes, and new behavioral challenges will provide a more comprehensive understanding of the broader impact of mTBI.

A major strength of this study is its multicenter, prospective design, which enables the capture of pre-injury functioning and timely follow-up of participants. Collecting baseline data from multiple informants—including patients, parents, and clinicians—will enhance the reliability and completeness of our data. Moreover, the use of validated instruments and standardized case report forms will support robust measurement across sites.

Our study is also designed to evaluate the representativeness of enrolled participants. By tracking eligible but non-enrolled patients and documenting basic demographic and clinical data, we will be able to assess potential enrollment bias—an important consideration in observational research. Additionally, our approach to risk stratification may lay the groundwork for future interventions, by identifying children at highest risk for poor mental health outcomes who may benefit from early referral or monitoring.

Nevertheless, our study will have limitations. As an observational study, we cannot establish causal relationships between risk factors and outcomes. There is also potential for loss to follow-up, which could introduce bias if those with worse outcomes complete follow-up assessments less frequently. To mitigate this, we have designed user-friendly methods of data collection, including the use of QR codes and brief validated surveys, and will make efforts to retain participants throughout the follow-up period.

In summary, our study will provide important insights into the trajectory of mental health following pediatric mTBI and help identify early indicators of risk. The findings have the potential to inform clinical care pathways and highlight opportunities for early intervention to support children recovering from mTBI.

### Study status

Participant enrollment for this study began on May 22, 2024. As of the time of manuscript submission, enrollment is ongoing at all participating sites. Baseline and follow-up data collection are actively underway, with follow-up assessments conducted at 1- and 3-months post-injury. The anticipated enrollment period is 4.5 years, with follow-up expected to conclude approximately three months after the final participant is enrolled. Data cleaning and preliminary analyses will begin following the completion of follow-up data collection. This is the first version of the study protocol.

## Supplementary Information

Supplementary Material 1.

## Data Availability

No datasets were generated or analysed during the current study.

## Abbreviations

ED: Emergency Department
GAD-7: Generalized Anxiety Disorder-7
IRB: Institutional Review Board
mTBI: Mild Traumatic Brain Injury
PedsQL: Pediatric Quality of Life Inventory
PHQ-8: Patient Health Questionnaire-8
SDQ: Strengths and Difficulties Questionnaire
STROBE: Strengthening the Reporting of Observational Studies in Epidemiology
