# Supplementary material for: Screening Emotions in Adolescents Receiving Care at the Hospital for mild Traumatic Brain Injury (SEARCH-mTBI): protocol for a multicenter observational study
Source: BMC Pediatr. 2026 Apr 7;26:450. doi: 10.1186/s12887-026-06788-5 (PMC13191971; doi:10.1186/s12887-026-06788-5)
Supplement: Supplementary file 1 — Supplementary Material 1. [file 12887_2026_6788_MOESM1_ESM.docx]

# Supplemental Material: STROBE checklist for the SEARCH-mTBI study

| **Section/Topic** | **STROBE Recommendation** | **Manuscript Location** |
| --- | --- | --- |
| Title and abstract | Indicate study design in title and provide informative abstract | Title, Abstract |
| Background/rationale | Explain scientific background and rationale | Background |
| Objectives | State specific objectives and hypotheses | Background |
| Study design | Present key elements of study design early | Methods: Study Design and Setting |
| Setting | Describe setting, locations, relevant dates | Methods: Study Design and Setting |
| Participants | Eligibility criteria, sources, and selection methods | Methods: Participants |
| Variables | Define all outcomes, exposures, predictors, confounders | Methods: Outcomes, Data Collection Procedures |
| Data sources/measurement | For each variable, give data sources and methods | Methods: Data Collections Procedures |
| Bias | Describe efforts to address potential sources of bias | Methods: Analysis |
| Study size | Explain how study size was determined | Methods: Sample Size |
| Quantitative variables | Explain handling of quantitative variables | Methods: Analysis |
| Statistical methods | Describe all statistical methods, including confounding control | Methods: Analysis |
| Participants (Results) | Report numbers at each stage of study | N/A - Results not yet available |
| Descriptive data | Characteristics of participants | N/A - Results not yet available |
| Outcome data | Report outcomes for each group | N/A - Results not yet available |
| Main results | Unadjusted and adjusted estimates, confounders | N/A - Results not yet available |
| Other analyses | Subgroup, interaction, sensitivity analyses | N/A - Results not yet available |
| Key results | Summarize key results with reference to objectives | Discussion |
| Limitations | Discuss limitations of study | Discussion |
| Interpretation | Cautious interpretation considering other evidence | Discussion |
| Generalisability | Discuss external validity of results | Discussion |
| Funding | Source of funding and role of funders | Declarations: Funding |
